# Sex-based differences in the manifestations and complications of sickle cell disease: Report from the Sickle Cell Disease Implementation Consortium

**DOI:** 10.1371/journal.pone.0258638

**Published:** 2021-10-29

**Authors:** Rita V. Masese, Dominique Bulgin, Mitchell R. Knisely, Liliana Preiss, Eleanor Stevenson, Jane S. Hankins, Marsha J. Treadwell, Allison A. King, Victor R. Gordeuk, Julie Kanter, Robert Gibson, Jeffrey A. Glassberg, Paula Tanabe, Nirmish Shah, on behalf of the Sickle Cell Disease Implementation Consortium

**Affiliations:** 1 School of Nursing, Duke University, Durham, North Carolina, United States of America; 2 College of Nursing, University of Tennessee Knoxville, Knoxville, Tennessee, United States of America; 3 RTI International, Research Triangle Park, North Carolina, United States of America; 4 Department of Hematology, St. Jude Children’s Research Hospital, Memphis, Tennessee, United States of America; 5 Department of Pediatrics, University of California San Francisco Benioff Children’s Hospital, Oakland, California, United States of America; 6 School of Medicine, Washington University in St. Louis, St. Louis, Missouri, United States of America; 7 University of Illinois at Chicago, Chicago, Illinois, United States of America; 8 University of Alabama, Birmingham, Alabama, United States of America; 9 Department of Emergency Medicine, Medical College of Georgia, Augusta University, Augusta, Georgia, United States of America; 10 Department of Emergency Medicine, Mount Sinai School of Medicine, New York, New York, United States of America; 11 School of Medicine, Duke University, Durham, North Carolina, United States of America; University of Cape Town, SOUTH AFRICA

## Abstract

**Introduction:**

Sex-based clinical outcome differences in sickle cell disease (SCD) remain largely unknown despite evidence that female sex is associated with an increased lifespan. To better characterize sex-based differences in SCD, we assessed pain, treatment characteristics, laboratory measures and complications among males and females currently enrolled in the Sickle Cell Disease Implementation Consortium (SCDIC) registry.

**Methods:**

The SCDIC consists of eight comprehensive SCD centers and one data coordinating center that received funding from the National Heart Lung and Blood Institute to improve outcomes for individuals with SCD. Eligibility criteria included: 15 to 45 years of age and a confirmed diagnosis of SCD. Self-report surveys were completed and data were also abstracted from the participants’ medical records.

**Results:**

A total of 2,124 participants were included (mean age: 27.8 years; 56% female). The majority had hemoglobin SS SCD genotype. Females had worse reports of pain severity (mean (SD) T-score 51.6 (9.6) vs 49.3 (10), p<0.001), more vaso-occlusive episodes (p = 0.01) and a higher occurrence of 3 or more hospital admissions in the past year (30.9% vs. 25.5, p = 0.03). On multivariable analysis, males had higher odds of acute chest syndrome (odds ratio (OR) 1.4, p = 0.002), cardiovascular (OR 1.70, p<0.001) and musculoskeletal (OR 1.33, p = 0.0034) complications and lower odds of depression (OR 0.77, p = 0.0381). Females had higher fetal hemoglobin levels with and without hydroxyurea use (9.6% vs 8.5%, p = 0.03 and 3% vs 2.2%, p = 0.0005, respectively).

**Conclusion:**

Our data suggests that sex differences in clinical outcomes do occur among individuals with SCD. Future research needs to explore the mechanisms underlying these differences.

## Introduction

Sickle cell disease (SCD) is a genetically inherited blood disorder that predominantly affects individuals of African descent [1,2]. One in 365 African Americans is born with SCD and 1 in 14 carry the trait [1,2]. The disease is characterized by polymerization of red blood cells into rigid sickle shapes during periods of low oxygen tension [3–6]. The disease is also associated with endothelial damage and chronic inflammation [3–6]. These factors result in vaso-occlusion and multi-organ dysfunction, leading to numerous complications such as vaso-occlusive pain episodes (VOEs), kidney failure and stroke [6].

Despite these complications, people with SCD are living longer [7]. In the United States, the median age of survival has increased from less than 20 years of age in the 1970s to 48 and 54.7 years of age for individuals with HbSS/HbSβ^0^ and Hb SC/HbSβ^+^ respectively, in the 2000s [1,8]. The increased median age of survival with SCD can be attributed to interventions to diagnose individuals with the disease much earlier in the life course (e.g., newborn screening) and interventions to prevent common disease-related complications (e.g., pneumococcal vaccination, use of penicillin prophylaxis, increased availability of hydroxyurea, and use of frequent blood transfusions for patients at increased risk of stroke) [9–13]. Of keen interest is that women live longer than their male counterparts [14].

Few studies have investigated sex-based SCD comparisons such as the frequency of VOEs, occurrence of end-organ dysfunction or complications, quality of life and sociodemographic characteristics [15–19]. The majority of those studies have been limited to urologic and obstetric disciplines [16–19]. A case series by Ballas et al [14] reviewed four women with SCD who lived beyond 80 years of age and linked the participants’ increased lifespan and quality of life to milder SCD, long-term family support, a healthy lifestyle and good adherence to medication and clinic appointments [14]. A prospective study by McClish et al [20] on gender differences in pain and healthcare utilization in SCD found an increase in VOEs and healthcare utilization (hospitalizations, doctor visits and ED visits) by men and no gender differences in opioid use and the frequency and intensity of pain [21]. Findings from this study, however, were limited by the small sample size and study scope. These underexplored differences may account for the sex difference in mortality. Therefore, a more comprehensive understanding of the sex differences in pain, treatment characteristics, laboratory measures and complications that exists amongst individuals with SCD is warranted.

To address this gap, we compared differences in sociodemographic and SCD disease characteristics and complications between men and women living with SCD currently enrolled in the Sickle Cell Disease Implementation Consortium (SCDIC) registry. Studying these differences may provide insight into the sex difference in mortality.

## Methods

### Study population

The study population included participants enrolled in the SCDIC patient registry. The SCDIC is a consortium, funded by the National Heart Lung and Blood Institute (NHLBI), including eight comprehensive sickle cell disease centers across the United States and one data coordinating center [22]. The overall goal of the SCDIC is to translate evidence-based SCD treatments to care [22]. One major priority of the consortium was the establishment of a comprehensive research registry that includes patient-reported and clinical data that can serve as a resource for conducting data queries and identifying gaps in research that inform implementation studies [22]. Participants were eligible for recruitment in the registry based on the following inclusion criteria: 15 to 45 years of age, confirmed diagnosis of SCD (subtypes Hb SS, SC, Sβ-thalassemia, SO, SD, SG, SE or SF), literacy in English and willingness to provide informed consent or assent. Confirmation of SCD diagnosis required laboratory confirmation (such as hemoglobin electrophoresis or newborn screening) of SCD from the participants’ medical records. Participants were excluded if they were unwilling or unable to provide informed consent or assent, had sickle cell trait (Hb AS), or had a successful bone marrow transplant. Recruitment occurred in outpatient clinics (e.g., sickle cell and primary care clinics), hospital inpatient settings, SCD support group meetings and conferences [23].

### Ethical approval

Ethical approval was received by the institutional review boards at each of the eight SCDIC study sites prior to any data collection efforts. Written informed consent was obtained before participant recruitment and enrollment in the study. For eligible participants younger than 18 years of age, informed consent was obtained from the parent or legal guardian in conjunction with informed assent from the participant. IRB approval was obtained for this analysis of the existing registry data from the Duke University Health System IRB (Protocol ID: Pro00103703). We analyzed partially anonymized data that was collected in the registry from July 2017 to February 2019. We accessed registry data in October 2019.

### Data collection

Data were collected using participant self-report surveys, medical records and laboratory abstraction forms (S1 Table) [24]. The data collection instruments were developed by the SCDIC steering committee, which consisted of at least one SCD expert from each of the eight sites [22]. The survey consisted of validated instruments that assessed socio-demographic information hydroxyurea use, opioid use, as well as pain frequency and severity [24]. Pain frequency and severity were assessed using five items from the pain episode frequency and severity domain of the Adult Sickle Cell Quality of Life Measurement Information System (ASCQ-Me) [24,25]. ASCQ-Me is a validated measure for assessing the health-related quality of life among people with SCD [24].

The medical records and laboratory forms collected data on the participants’ SCD genotype, blood transfusion history, number of VOEs in the past year, specialists involved in care, number of hospital admissions in the past year, SCD complications and standard laboratory measures (S1 Table) [24].

Data for the medical and laboratory forms were abstracted from participants’ electronic health records. Laboratory measures were obtained during steady state, which was defined as at least two weeks before or after: 1) hospitalization, 2) a blood transfusion, or 3) a major acute event (e.g., stroke or VOE) [24]. Survey and abstracted health record data were entered into a REDCap database, assessed for completeness and inaccuracies by the data coordinating center, and referred back to each site for correction.

### Data analysis

We utilized a cross-sectional study design. Male participants were compared to female participants with regards to sociodemographic characteristics, SCD disease characteristics and complications. Summary statistics were presented as frequencies and percentages for categorical variables, and median and 25^th^– 75^th^ percentiles (Q1-Q2) or mean and standard deviation for continuous variables. Categorical variables were analyzed using Chi-Square or Fisher exact tests when appropriate. Continuous variables were compared using the Mann-Whitney U test or independent sample t-tests depending on the distribution. For each SCD complication, all variables with p ≤ 0.1 in univariate analysis were included in a multivariable logistic regression with backward elimination. Statistical analysis was performed using SAS version 9.4 (SAS Institute; Cary, NC). A p-value < 0.05 was considered statistically significant. Due to the exploratory and hypothesis generating approach of the study, no adjustment for multiple testing was applied [26].

## Results

### Demographic characteristics

**Fig 1:**
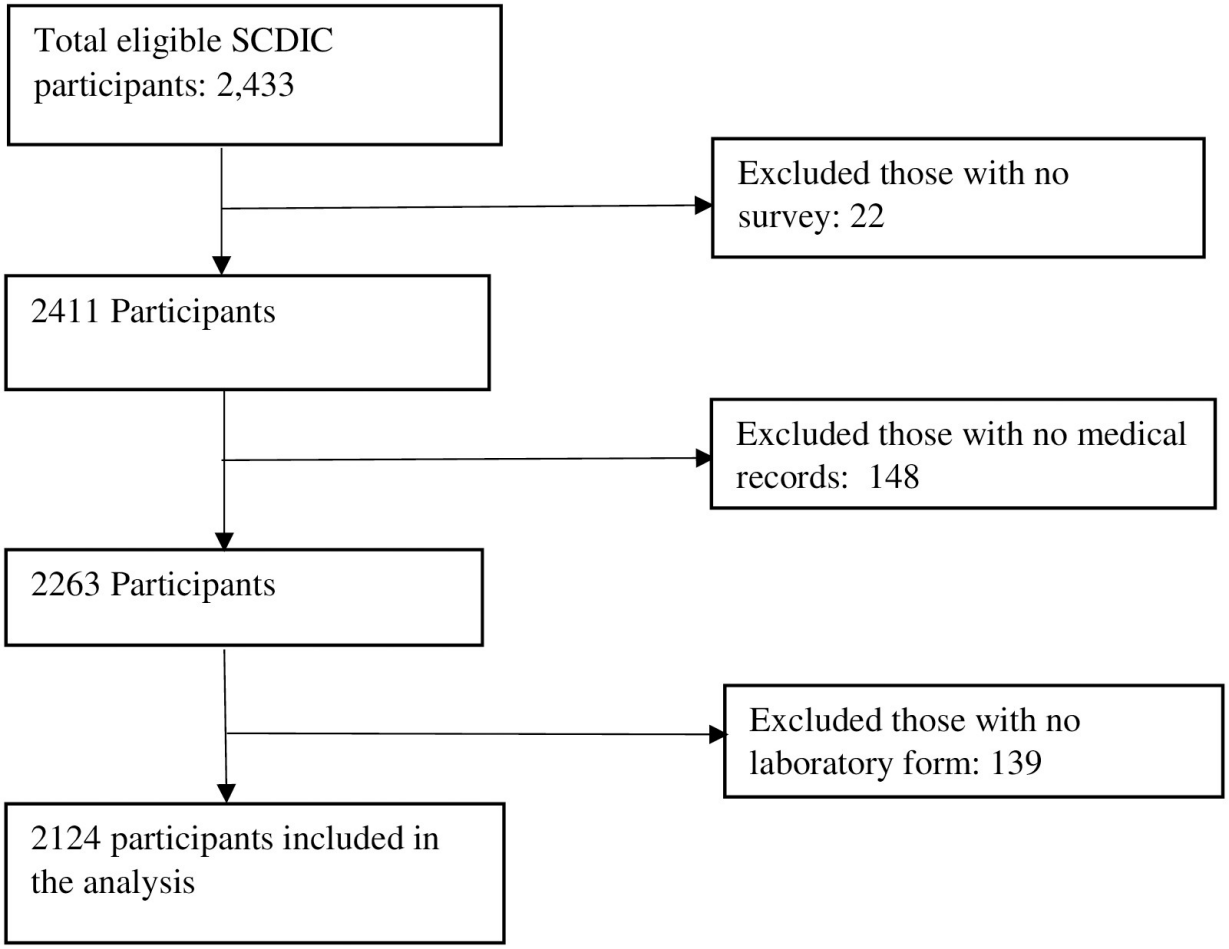
Flow diagram of participant inclusion.

A total of 2124 participants were included in the study; 1190 were female (Fig 1).

Participant demographics are highlighted in Table 1. The mean age of our participants was 27.8 years. The majority were Black or African American (95.6%) and never married (78.4%). There were no statistical differences in the age, ethnicity, race, employment, and marital status between sexes. Females had higher levels of education (25.9% vs 16.8% college graduate and 83.6% vs. 79.7% high school education and higher, p <0.0001), but had a household income less than males (p = 0.04). Although not statistically significant, more women were on government insurance (i.e. Medicare, Medicaid, or other government sponsored programs).

**Table 1:** Participant socio-demographic characteristics.

Characteristic	Females (n = 1190)	Males (n = 934)	P-value	Total (n = 2124)
**Age**				
Mean (SD) years	27.9 (7.8)	27.6 (8.0)	0.3	27.8 (7.9)
Median (Q1-Q2)	27 (22–34)	27 (21–33)		27 (21–34)
**Race, N (%)**				
Black or African American	1,128 (96.6)	856 (94.3)	0.1	1,984 (95.6)
Multiracial	28 (2.4)	41 (4.5)		69 (3.3)
American Indian/Alaskan	6 (0.5)	4 (0.4)		10 (0.5)
Asian	3 (0.3)	3 (0.3)		6 (0.3)
White	3 (0.3)	4 (0.4)		7 (0.3)
**Ethnicity, N (%)**				
Hispanic/ Latino	44 (3.8)	45 (4.9)	0.2	89 (4.3)
**Marital Status, N (%)- adults only**				
Never married	827 (79.1)	614 (77.3)	0.1	1,441 (78.4)
Married/Living as married	162 (15.5)	129 (16.2)		291 (15.8)
Divorced/separated/widowed	56 (5.4)	51 (6.4)		107 (5.8)
**Education, N (%)**				
Less than high school	31 (2.6)	33 (3.6)	<0.0001	64 (3.1)
Some high school	161 (13.7)	152 (16.6)		313 (15.0)
High school graduate or GED equivalent	285 (24.3)	305 (33.4)		590 (28.3)
Some college	391 (33.4)	270 (29.5)		661 (31.7)
College graduate	303 (25.9)	154 (16.8)		457 (21.9)
**Employment, N (%)**				
Employed	413 (35.5)	339 (37.4)	0.3	752 (36.3)
Not employed by choice	286 (24.6)	198 (21.9)		484 (23.4)
Not employed, other	464 (39.9)	369 (40.7)		833 (40.3)
**Household income in a year, N (%)**				
$25,000 or less	610 (56.5)	412 (50.6)	0.04	1,022 (54.0)
$25,001-$50,000	234 (21.7)	194 (23.8)		428 (22.6)
$50,001-$75,000	98 (9.1)	103 (12.6)		201 (10.6)
$75,001-$100,000	55 (5.1)	48 (5.9)		103 (5.4)
>$100,001	82 (7.6)	58 (7.1)		140 (7.4)
**Medical Insurance, N (%)**				
None	48 (4.0)	47 (5.0)	0.2	95 (4.5)
Medicare, Medicaid or government-sponsored	826 (69.4)	618 (66.2)		1,444 (68.0)
Private	316 (26.6)	268 (28.7)		584 (27.5)

Missing values were not included in the comparison. The p-values reported represent strength of associations between variables.

### Disease and treatment characteristics

Most participants (male and female) had the hemoglobin SS genotype and received care from hematologists. The prevalence of hydroxyurea use was higher in males (55.4% vs. 44.6%, p <0.0001) (Table 2). Females had worse ASCQ-Me pain frequency and severity scores (p = 0.0002 and <0.0001, respectively) and had a higher rate of 3 or more admissions in the past year (30.9% vs 25.5, p = 0.03) (Table 2). Females and males had similar rates of taking opioids (80.4% vs. 78.9%). Males had significantly more skin ulcers and respiratory, musculoskeletal, genitourinary and cardiovascular complications. In contrast, females were more likely to have anxiety, depression and autoimmune diseases.

**Table 2:** Disease characteristics.

Characteristic	Females (N = 1190)	Males (N = 934)	P-value
**Sickle cell genotype, N (%)**			
Hb SS	805 (67.7)	642 (68.7)	0.4
Hb SC	255 (21.4)	194 (20.8)	
Hb S beta+ thalassemia	75 (6.3)	45 (4.8)	
Hb S beta0 thalassemia	40 (3.4)	43 (4.6)	
Other variants	14 (1.2)	10 (1.1)	
**Hydroxyurea use, N (%)**			
Currently using	519 (44.6)	511 (55.4)	<0.0001
**Opioid Use, N (%)**			
Currently using opioids	957 (80.4)	737 (78.9)	0.4
**Number of vaso-occlusive episodes in the past year, N (%)**			
0	140 (11.8)	139 (15.0)	0.01
1	109 (9.2)	109 (11.7)	
2	151 (12.7)	134 (14.4)	
3	196 (16.5)	147 (15.8)	
4 or more	590 (49.7)	400 (43.1)	
**Time since most recent vaso-occlusive episode, N (%)**			
Currently having one	150 (12.6)	108 (11.6)	0.1
<1 week ago	204 (17.2)	127 (13.6)	
1–3 weeks ago	255 (21.5)	189 (20.3)	
1–6 months ago	319 (26.9)	257 (27.6)	
7–11 months ago	73 (6.2)	63 (6.8)	
1–5 years ago	119 (10.0)	120 (12.9)	
5+ years ago	47 (4.0)	46 (4.9)	
Never had a pain attack	19 (1.6)	21(2.3)	
ASCQ-Me Pain Episode frequency T-score, mean (SD)	49.2 (11.1)	47.4 (11.7)	0.0002
ASCQ-Me Pain Episode severity T-score, mean (SD)	51.6 (9.6)	49.3(10)	<0.001
**Prior History of Blood Transfusion, N (%)**	1195 (64.0)	672 (65.4)	0.2
**Number of hospital admissions in the past year, N (%)**			
0	344 (35.0)	318 (41.6)	0.03
1	215 (21.9)	172 (22.5)	
2	120 (12.2)	80 (10.5)	
3	84 (8.6)	50 (6.5)	
4+	219 (22.3)	145 (19.0)	
**Specialists involved in care, N (%)**			
PCP only	26 (2.2)	8 (0.9)	0.049
Hematologist only	700 (59.4)	579 (62.9)	
Co-management (PCP & Hematologist)	442 (37.5)	329 (35.7)	
Other	10 (0.8)	5 (0.5)	
**Sickle cell disease related complications, N (%)**			
Acute chest syndrome	658 (55.4)	581 (62.3)	0.001
Asthma	319 (26.9)	253 (27.1)	0.9
Digestive^a^	729 (61.3)	544 (58.4)	0.1
Musculoskeletal^b^	351 (29.5)	332 (35.6)	0.003
Autoimmune/Inflammatory^c^	228 (19.6)	141 (15.5)	0.01
Central nervous system^d^	200 (16.8)	162 (17.3)	0.7
Genitourinary^e^	84 (7.1)	304 (32.6)	<0.001
Cardiovascular^f^	138 (11.6)	160 (17.1)	0.0003
Multi-organ failure	62 (5.2)	49 (5.2)	0.9
Pneumococcal sepsis	40 (3.4)	47 (5.0)	0.06
Skin ulcers	33 (2.8)	54 (5.8)	0.0005
Retinopathy	165 (13.9)	145 (15.5)	0.3
Diabetes mellitus	28 (2.4)	20 (2.1)	0.7
Iron overload	308 (25.9)	226 (24.2)	0.4
Chronic refractory pain	263 (22.1)	184 (19.7)	0.2
Anxiety	174 (14.6)	104 (11.1)	0.02
Depression	259 (21.8)	154 (16.5)	0.002
Cancer	4 (0.3)	4 (0.4)	0.7

Missing values were not included in the comparison. The p-values reported represent strength of associations between variables.

^a^Digestive complications: splenomegaly, splenic sequestration, splenic infarcts, hypersplenism, autosplenectomy, gallstones and cholecystitis.

^b^Musculoskeletal complications: dactylitis, avascular necrosis and osteomyelitis.

^c^Autoimmune/Inflammatory complications: deep venous thrombosis, lupus, rheumatoid arthritis, gout and sarcoidosis.

^d^Central nervous system complications: stroke and intracranial bleeding.

^e^Genitourinary complications: priapism, chronic kidney disease and end stage renal failure.

^f^Cardiovascular complications: pulmonary arterial hypertension and left ventricular dysfunction.

On multivariable analysis, males had higher odds of acute chest syndrome (odds ratio (OR) 1.4, p = 0.002; Table 3), cardiovascular (OR 1.70, p<0.001) and musculoskeletal (OR 1.33, p = 0.0034) complications and lower odds of depression (OR 0.77, p = 0.0381). Unemployed females had higher odds of anxiety (OR = 1.72, p = 0.0049).

**Table 3:** Association between participant demographics and sickle cell disease complications.

Variable	P value	OR (95% CI)
**Acute chest syndrome**		
**Age**	< .0001	0.96 (0.95–0.97)
**Sex**	0.002	
Female (ref)		
Male		1.4 (1.14–1.73)
**Hydroxyurea use**	0.05	
No (Ref)		
Yes		0.8 (0.64–0.99)
**Medical insurance**	< .0001	
Private (Ref)		
None		0.92 (0.54–1.55)
Medicare, Medicaid or government-sponsored		1.8 (1.44–2.27)
**SCD genotype**	< .001	
Hb SC (Ref)		
Hb SS		1.73 (1.33–2.26)
Hb S beta+ thalassemia		0.59 (0.35–0.96)
Hb S beta0 thalassemia		2.04 (1.14–3.7)
Other variants		1.04 (0.41–2.6)
**Cardiovascular complications**		
**Age**	< .0001	1.06 (1.04–1.08)
**Sex**	< .0001	
Female (Ref)		
Male		1.70 (1.32–2.21)
**Medical Insurance**	< .0001	
Private (Ref)		
None		1.17 (0.46–2.56)
Medicare, Medicaid or government-sponsored		1.93 (1.40–2.72)
**SCD genotype**	0.003	
Hb SC (Ref)		
Hb SS		2.68 (1.82–4.09)
Hb S beta+ thalassemia		0.95 (0.37–2.14)
Hb S beta0 thalassemia		1.82 (0.77–3.93)
Other variants		1.79 (0.40–5.72)
**Musculoskeletal complications**		
**Age**	< .0001	1.05 (1.03–1.06)
**Sex**	0.0034	
Female (Ref)		
Male		1.33 (1.10–1.62)
**SCD genotype**	0.006	
Hb SC (Ref)		
Hb SS		1.57 (1.21–2.05)
Hb S beta+ thalassemia		1.05 (0.64–1.70)
Hb S beta0 thalassemia		1.46 (0.85–2.46)
Other variants		0.81 (0.26–2.12)
**Hydroxyurea current use**	0.0083	
No (Ref)		
Yes		0.76 (0.63–0.93)
**Employment**	0.0006	
Employed (Ref)		
Not employed by choice		1.63 (1.23–2.15)
Not employed, other		1.41 (1.13–1.76)
**Depression**		
**Age**	0.02	1.02 (1.00–1.04)
**Sex**	0.0381	
Female (Ref)		
Male		0.77 (0.60–0.99)
**Medical Insurance**	0.03	
Private (Ref)		
None		0.56 (0.23–1.20)
Medicare, Medicaid or government-sponsored		1.33 (0.98–1.82)
**Employment**	0.02	
Not employed, other (Ref)		
Employed	0.514	1.14 (0.77–1.66)
Not employed by choice	0.006	1.50 (1.12–2.00)
**Anxiety**		
Employment*sex	0.02	
Not employed by choice vs Employed gender = Female	0.8811	0.97 (0.60–1.54)
Not employed, other vs Employed gender = Female	0.0049	1.72 (1.18–2.51)
Not employed by choice vs Employed gender = Male	0.1717	1.46 (0.85–2.51)
Not employed, other vs Employed gender = Male	0.9066	1.03 (0.63–1.69)

Model includes all variables with p<0.1 in univariate analysis.

Interaction between sex and other covariates were not significant and were eliminated from the final model.

Concerning laboratory measures, males had significantly higher blood urea nitrogen (BUN), serum creatinine and liver enzymes (aspartate transaminase (AST), alanine transaminase (ALT) and alkaline phosphatase (ALP) and albumin (Table 4). Reticulocyte count, white blood cell count and differentials (neutrophils and monocytes) were similar in both sexes. Males had significantly higher mean steady state hemoglobin (10 g/dl vs 9.3 g/dl, p<0.0001). Female participants had significantly higher fetal hemoglobin levels with and without hydroxyurea use (9.6% vs 8.5%, p = 0.03 and 3% vs 2.2%, p = 0.0005, respectively).

**Table 4:** Laboratory measures.

Laboratory measure	Females		Males		P-value
	n	Median or mean (Q1-Q2 or SD)	n	Median or mean (Q1-Q2 or SD)	
Hemoglobin g/dl, mean (SD)	1183	9.3 (1.70)	930	10.0 (2.10)	<0.0001
Hb A2%, median (Q1-Q2)	921	3.5 (3.0–4.1)	725	3.6 (3.0–4.5)	0.1
Hb F % with hydroxyurea use, median (Q1-Q2)	445	9.6 (5–16.9)	451	8.5 (3.5–16)	0.03
Hb F % without hydroxyurea use, median (Q1-Q2)	521	3 (1.3–8)	306	2.2 (1–5.4)	0.0005
Platelet count 10^9^/l, mean (SD)	1180	345 (141.0)	926	337 (149.0)	0.2
MCV fl, mean (SD)	1179	88.9 (13.5)	927	90.1 (14.5)	0.07
MCH pg, mean (SD)	1182	31.0 (5.9)	928	31.5 (5.6)	0.1
MCHC g/dl, mean (SD)	1175	34.6 (1.5)	925	34.7 (1.6)	0.1
Reticulocyte Count 10^3^/mm^3^, median (Q1-Q2)	588	0.5 (0.1–2)	439	0.5 (0.1–2)	0.15
White Blood Cells 10^3^/mm^3^, mean (SD)	1180	10.6 (4.5)	929	10.1 (4.1)	0.01
Neutrophils segmented and band %, mean (SD)	1116	55.9 (12.5)	889	53.4 (12.8)	<0.0001
Lymphocytes %, mean (SD)	1128	31.7 (11.4)	902	31.9 (11.8)	0.7
Monocytes %, mean (SD)	1127	8.5 (4.5)	898	9.8 (5.1)	<0.0001
Serum BUN mg/dL, median (Q1-Q2)	1155	7.0 (5.0–9.0)	910	9.0 (7–11)	<0.0001
Serum Creatinine mg/dL, median (Q1-Q2)	1158	0.6 (0.5–0.7)	916	0.8 (0.6–0.9)	<0.0001
Bilirubin serum total mg/dL, median (Q1-Q2)	1153	2.0 (1.2–3.2)	912	2.4 (1.5–4.1)	<0.0001
Bilirubin serum direct mg/dL, median (Q1-Q2)	602	0.4 (0.3–0.7)	460	0.4 (0.3–0.7)	0.8
AST U/L, median (Q1-Q2)	1150	31.0 (22.0–45.0)	907	35.0 (26.0–49.0)	<0.0001
ALT U/L, median (Q1-Q2)	1150	19.0 (13.0–29.0)	908	22.0 (15.0–31.0)	<0.0001
Alkaline Phosphatase U/L, median (Q1-Q2)	1153	75.0 (60.0–100.0)	907	89.0 (70.0–116.0)	<0.0001
Total Protein g/dL, mean (SD)	1146	7.6 (0.70)	906	7.7 (0.7)	0.07
Albumin g/dL, mean (SD)	1146	4.1 (0.50)	902	4.3 (0.5)	<0.0001

Missing values were not included in the comparison. The p-values reported represent strength of associations between variables.

## Discussion

This study identified important sex differences in disease characteristics and complications. In this cohort of 2124 people, females had higher fetal hemoglobin levels despite reporting less hydroxyurea use than males. Females also had more opioid use, significantly worse ASCQ-Me pain frequency and severity scores, and more VOEs and hospitalizations. Despite more hydroxyurea use and less hospitalizations, males had more life-threatening complications; while females had higher rates of anxiety, depression and autoimmune diseases.

Recurrent occurrences of VOEs are the hallmark presentation of SCD and are the leading cause of hospitalization [27]. Almost half of our participants reported having four or more VOEs in the past year, majority of whom were female. A prospective cohort study on life expectancy and risk factors for early death in SCD by Platt et al [1] revealed that patients with SCD have an average of two or more VOEs per year in the absence of treatment [1]. In our study, females reported worse ASCQ-Me pain frequency and severity scores and were taking more opioids. The higher pain and opioid usage in females may be explained by previous literature which identified an increase in VOEs at different stages of the menstrual cycle [28–30]. In addition to the increase of VOEs with menstruation, a study by Brandow et al [31] revealed that females with SCD expressed more neuropathic pain than males [31]. Further investigations on the assessment of pain and the influence of hormonal and physiological changes related to menstruation and the impact they may have on the frequency of VOEs in females with SCD are warranted.

In our registry, more males self-reported taking hydroxyurea than females; however, females had higher levels of fetal hemoglobin. Hydroxyurea use increases levels of fetal hemoglobin and has been associated with a reduction in blood transfusion dependency and mortality in SCD [32]. In addition, higher fetal hemoglobin levels has been associated with increased survival in SCD [1]. Our finding of elevated fetal hemoglobin in females, with and without hydroxyurea use, may contribute to the increased survival in women with SCD. Further studies are needed to determine other causes of elevated fetal hemoglobin in females. Despite the elevated fetal hemoglobin, females reported lower hydroxyurea use. Reasons for lower hydroxyurea use among females identified in the literature include the proven teratogenic effects of the drug in animals; which deters use during pregnancy [33] and has led to recommendations to discontinue hydroxyurea when attempting to become pregnant, during pregnancy or while breast feeding [13]. A key consideration that needs to be taken into account in the interpretation of our findings is that our data on hydroxyurea was self-reported and lacks indicators of adherence. The possibility that males self-reported hydroxyurea use, but may not be adherent cannot be ruled out. Additional investigations into reasons for less hydroxyurea use in females and the association between self-reported hydroxyurea use and adherence in SCD populations are warranted.

Except for autoimmune diseases, females had significantly less prevalence of chronic end-organ complications than their male counterparts [34]. A study by Ngo et al [34] revealed a higher prevalence of autoimmune diseases in females compared to males [34]. The higher prevalence may be attributed to gender specific hormones and the role of those hormones during the various reproductive stages of a woman [34]. Flare up of autoimmune diseases such as systemic lupus erythematosus are influenced by the age of menarche, menopause or the use of oral contraceptives [34]. Gender-specific hormones may account for the higher occurrence of autoimmune complications among females in our registry.

Males had significantly more complications associated with increased mortality, including skin ulcers, acute chest syndrome, musculoskeletal, genitourinary and cardiovascular complications. Our findings are also consistent with Platt et al [1] findings that males have a lower median age at death than females and individuals with symptomatic SCD have higher mortality [1]. For instance, respiratory complications, such as acute chest syndrome, are the leading cause of morbidity and mortality amongst individuals with SCD [35], and more males in our sample had a diagnosis of acute chest syndrome. Males in our sample also had significantly more skin ulcers than females, which has also been previously reported [36]. The higher odds of SCD complications in males could be associated with the higher lab values in males, such as liver enzymes. Although lab values were within normal ranges, this may suggest a higher pro-inflammatory state among men, which has been well reported in SCD [37,38]. Close monitoring of lab values, particularly among males, is warranted to screen for and prevent the occurrence of disease complications that lead to early mortality. Our results support the need for further research to uncover the reasons for a higher incidence of chronic end-organ damage amongst males with SCD and determine if specific social and medical interventions may improve the lifespan of males living with SCD.

Importantly, females had more frequent diagnoses of anxiety and depression than males, consistent with the general population [39,40]. In 2008, using data from the Pain in Sickle Cell Epidemiology Study (PiSCES) study, Levenson et al [41] found that individuals with SCD reporting depressive symptoms and high anxiety had significantly more incidence of pain and also experienced more distress and interference from pain in their daily lives [41]. This is consistent with our findings that females with SCD had significantly more frequent and severe pain in addition to depression and anxiety diagnoses compared to males with SCD. Depression and anxiety is known to exacerbate pain and other physical complications of SCD [41]. In addition to impacts on physical and mental quality of life, depression has also been associated with a significant increase in total health care costs for individuals with SCD ($13,016 for no depression diagnosis vs $30,665 for depression diagnosis p = 0.01) [42]. Despite higher rates of depression and anxiety, we found females were more likely to have higher education. Interestingly, we also found females reported a lower household income than male participants with SCD. Income inequality has been associated with an increased risk of depression [43].

Our study has several limitations. All the individuals with SCD that participated in the study were recruited through comprehensive SCD care settings where they receive care from sickle cell specialists. Thus, this sample is not representative of individuals who do not have access to SCD experts. Only individuals that had literacy in English were included and our findings are not generalizable to individuals who have low literacy levels or who are non-English speaking. Next, we did not collect information on psychosocial factors (e.g. social support) and lifestyle factors (e.g. smoking, alcohol, diet, exercise) that are linked to quality of life and lifespan [20,44–46]. We were also unable to account for the impact that gender socialization may have on individuals’ behaviors, such as disease self-management. For instance, prior studies have associated femininity with engagement in health promotion [47]. Findings from a recent study by Blake et al [48] suggest that health promotion in SCD is linked to gender socialization; as parents or parental proxies of adolescents with SCD tended to underestimate their male adolescents emotional and social functioning, while overestimating for female adolescents.^46^ Finally, this study is limited due to the use of a cross-sectional design. The timing of individuals completing the surveys and having their medical records abstracted may not be representative of the sex-based differences in the manifestations and complications of SCD that exists across the lifespan. We abstracted data entered by healthcare providers in the participant’s medical records and could not fully ascertain that there was a gender differential in the measurements and recording of those data in the participants’ medical records.

Despite those limitations, our study has a large sample size and utilizes data from a consortium with eight geographically diverse comprehensive SCD care settings and continues to follow these participants through the prospective registry. Our sample size had sufficient power to detect statistically significant differences between males and females. Additionally, validated instruments were used to collect self-report data.

## Conclusion

Our findings suggest key sex differences in the presentation of SCD, with males having more life-threatening chronic end-organ complications and females having higher rates of depression and anxiety. Future research is required to determine how sex influences the mechanisms underpinning clinical outcome differences.

## Supporting information

S1 Table Data variables. (DOCX)
