# Supplementary material for: Sex-based differences in the manifestations and complications of sickle cell disease: Report from the Sickle Cell Disease Implementation Consortium
Source: PLoS One. 2021 Oct 29;16(10):e0258638. doi: 10.1371/journal.pone.0258638 (PMC8555833; doi:10.1371/journal.pone.0258638)
Supplement: S1 Table — (DOCX) [file pone.0258638.s001.docx]

**S1 Table. Data variables**

| Variable | Self-report survey | Medical Record |
| --- | --- | --- |
| Sociodemographic data (i.e., age, sex, race, ethnicity, household income, educational attainment, marital status) | X |  |
| Insurance status |  | X |
| Sickle cell disease genotype | X |  |
| Frequency & severity of pain  (ASCQ-Me Pain episode Frequency and Pain Episode Severity item bank) | X |  |
| Medication History: Hydroxyurea and Opioid use  (ASCQ-Me Medical History Checklist) | X |  |
| Blood Transfusion History |  | X |
| Hospital visits |  | X |
| Specialists involved in care |  | X |
| SCD complications^a^  1. Respiratory: asthma, acute chest syndrome.  2. Digestive: splenomegaly, splenic sequestration, splenic infarcts, hypersplenism, autosplenectomy, gallstones, cholecystitis.  3. Musculoskeletal: dactylitis, avascular necrosis, osteomyelitis.  4. Autoimmune/Inflammatory: deep venous thrombosis, lupus, rheumatoid arthritis, gout, sarcoidosis.  5. Central nervous system: stroke, intracranial bleeding.  6. Genitourinary: priapism, chronic kidney disease, end stage renal failure.  7. Cardiovascular: Pulmonary arterial hypertension, left ventricular dysfunction. |  | X |
| Laboratory Measures^b^:  hemoglobin, platelet count, mean cell volume (MCV), mean cell hemoglobin concentration (MCHC), white blood cell count and differential (neutrophils, lymphocytes, monocytes), serum blood urea nitrogen (BUN), serum creatinine, serum bilirubin, alanine aminotransferase (ALT), aspartate aminotransferase (AST), alkaline phosphatase, total protein, albumin |  | X |

^a^ SCD complications outlined were treated as such in the analysis

^b^ Most recent value obtained at steady state i.e. two weeks before or after hospitalization, blood transfusion or a major acute event.
